# Clinical applications of circulating tumor cells in hepatocellular carcinoma

**DOI:** 10.3389/fonc.2022.968591

**Published:** 2022-08-24

**Authors:** Yinggang Hua, Jingqing Dong, Jinsong Hong, Bailin Wang, Yong Yan, Zhiming Li

**Affiliations:** ^1^ Department of General Surgery, Guangzhou Red Cross Hospital, Jinan University, Guangzhou, China; ^2^ Institute of Reproductive Health, Tongji Medical College, Huazhong University of Science and Technology, Wuhan, China

**Keywords:** circulating tumor cells, hepatocellular carcinoma, epithelial-mesenchymal transformation, clinical application, detection methods

## Abstract

Hepatocellular carcinoma (HCC) is a highly malignant tumor and ranked as the fourth cause of cancer-related mortality. The poor clinical prognosis is due to an advanced stage and resistance to systemic treatment. There are no obvious clinical symptoms in the early stage and the early diagnosis rate remains low. Novel effective biomarkers are important for early diagnosis and tumor surveillance to improve the survival of HCC patients. Circulating tumor cells (CTCs) are cancer cells shed from primary or metastatic tumor and extravasate into the blood system. The number of CTCs is closely related to the metastasis of various solid tumors. CTCs escape from blood vessels and settle in target organs, then form micro-metastasis. Epithelial-mesenchymal transformation (EMT) plays a crucial role in distant metastasis, which confers strong invasiveness to CTCs. The fact that CTCs can provide complete cellular biological information, which allows CTCs to be one of the most promising liquid biopsy targets. Recent studies have shown that CTCs are good candidates for early diagnosis, prognosis evaluation of metastasis or recurrence, and even a potential therapeutic target in patients with HCC. It is a new indicator for clinical application in the future. In this review, we introduce the enrichment methods and mechanisms of CTCs, and focus on clinical application in patients with HCC.

## Introduction

Hepatocellular carcinoma (HCC) accounts for about 90% of all primary hepatic malignancies and is one of the most common malignancies. HCC ranks as the sixth most common tumor and its mortality ranks fourth in cancer-related death worldwide, with a high incidence in Asia and Africa (1). Patients with early-stage HCC may undergo curative therapies such as surgical liver resection (LR), liver transplantation (LT) and local ablation. On the other hand, patients with advanced-stage HCC usually accept non-curative therapies such as transcatheter arterial chemoembolization, radiotherapy and systemic therapies (2). Due to the absence of obvious clinical symptoms in the early stage of HCC, most patients are diagnosed with advanced HCC for the first time. The existing methods are difficult to effectively prolong the survival of patients, with a 5-year survival rate of less than 20%. Therefore, the key to improving the prognosis of HCC patients are to find effective means for early diagnosis, surveillance response of treatment, and early intervention (3).

Currently, the diagnosis of HCC mainly depends on serum markers, imaging examination and liver biopsy. Serum alpha-fetoprotein (AFP) is the most widely used tumor marker for early screening and surveillance progression of HCC, nevertheless, it has unsatisfactory performance with a sensitivity of only 60% and specificity of only 80% (4, 5). The updated American Association for the Study of Liver Disease (AASLD) guidelines no longer recommended AFP testing as part of HCC diagnostic criteria (6). Other serum markers, such as osteopontin, Golgi protein-73 or glypican-3 may offer information about the biological aggressiveness of HCC, but they are not erratic and accurate enough to form part of a screening strategy (7–9). Medical imaging methods including B-ultrasound, computed tomography (CT), and magnetic resonance imaging (MR) are recommended for the diagnosis of HCC, however, those methods are difficult to detect early-stage liver cancer with a diameter of less than 1 cm. Although liver biopsy can provide definitely a pathological diagnosis, there is a debate on the widespread use of liver biopsy due to the risks of bleeding and tumor seeding (10). Therefore, we need to find effective markers for early diagnosis and monitoring of recurrence and metastasis in patients with HCC.

In recent years, liquid biopsy technology has gradually emerged and has become one of the most promising methods for early diagnosis and real-time progress assessment of tumors with the advantages of non-invasive and repeated sampling. CTCs, which are the most concerned tumor detection method in liquid biopsy, is obtained from the peripheral blood of patients and carry much comprehensive tumor information for early diagnosis and monitoring of tumors. CTCs were originally discovered in the blood of breast cancer patients by Australian physicians Thomas Ashworth (11). CTCs are a very scarce sub-population of cancer cells released from primary solid tumors or metastatic sites into the peripheral circulation and eventually form metastatic lesions in other target organs (12). The number of CTCs in the circulating system is extremely rare (only a few CTCs in billions of blood cells), a range of 0 − 86 CTCs were detected in 5 mL of blood in HCC patients (13).

In the early stages, little is known about the characteristics and phenotypes of CTCs, and the limitation of enrichment technique leads to a big challenge for the detection of CTCs. With the emergence of new technologies, the improvement of existing technologies and the deepening of understanding of oncology, the detection rate of CTCs has been greatly improved. Currently, CTCs detection has been applied in clinical trials on a small scale. The CellSearch system for CTCs detection, the only approved technology by the US Food and Drug Administration, has been used for breast, lung, prostate and colon cancer (14–16). In recent years, CTCs detection plays an increasingly important role in HCC clinical management.

CTCs can not only provide information about abnormal protein expression, genomic mutation and mRNA variation of solid tumors, but also help people understand the mechanism of tumorigenesis, metastasis and drug resistance from aspects of cell morphology, migration ability and drug response (17–19). Correspondingly, CTCs detection can be used for early diagnosis, individualized treatment, and monitoring of prognosis and recurrence (20). In HCC, rich blood is present around the immediate vicinity of the tumor, allowing thousands of CTCs to be released into the blood circulation daily. CTCs carry a large amount of tumor information and are used as a clinical biomarker for HCC. In the present review, we introduce the enrichment methods and different phenotypes of CTCs and focus on clinical application in patients with HCC.

## Enrichment and identification of CTCs

About 10^6^ CTCs fall off into the peripheral blood circulation per gram of tumor primary or metastatic tissue daily, but more than 99.99% of CTCs lose their activity under the attack of the human immune defense system, and only <0.01% of CTCs can survive (21). Every milliliter of blood contains only a few of CTCs, while there are billions of normal blood cells, various proteins, nucleic acids, carbohydrates and other substances. The sensitivity of the enrichment effect is greatly affected by the background interference. Most of the CTCs travel as individual cell, but some as clusters, also known as micro-emboli. Micro-emboli are more easily infiltrate distant tissues and form metastases than individual cell in cancers (22–24). In addition, some CTCs can interact with platelets in the blood and accomplish the phenotypic transformation of tumor cells, resulting in poor prognosis (25, 26). A series of techniques recently have been developed to separate and enrich CTCs from complex background. The capture methods can be divided into the physical techniques and biological techniques according to the properties of CTCs.

Physical methods capture CTCs from erythrocytes and leukocytes through the physical properties of CTCs, including size, density, deformability, and electrical charge. Density gradient centrifugation is based on the difference in density and sedimentation rate between CTCs and blood cells. CTCs and blood components are distributed to specific positions under a certain centrifugal force (27). This method is simple to operate, but is not widely used due to its low sensitivity, specificity and time-consuming. Microfiltration is a physical method based on the assumption that the diameter of tumor cells is too larger than erythrocytes and leukocytes to pass through a filter with lots of small pores (28, 29). However, some studies showed the cell-size based microfiltration loss amount of small size CTCs, resulting in false negative results (30–32). The CTCs ≤ 5μm are particularly observed in a majority of HCC CTCs, which is lost during microfiltration (30). Dielectrophoresis is a method to separate CTCs from blood according to the difference of charge between CTCs and other blood cells (33). Other physical separation and enrichment techniques include microfluidic, acoustophoresis, et al. (34, 35). CTCs enrichment based on physical properties enables high-throughput, but these methods possess a low specificity and a higher false-positive rate due to some blood cells may exhibit similar physical properties to CTCs.

**Figure 1:**
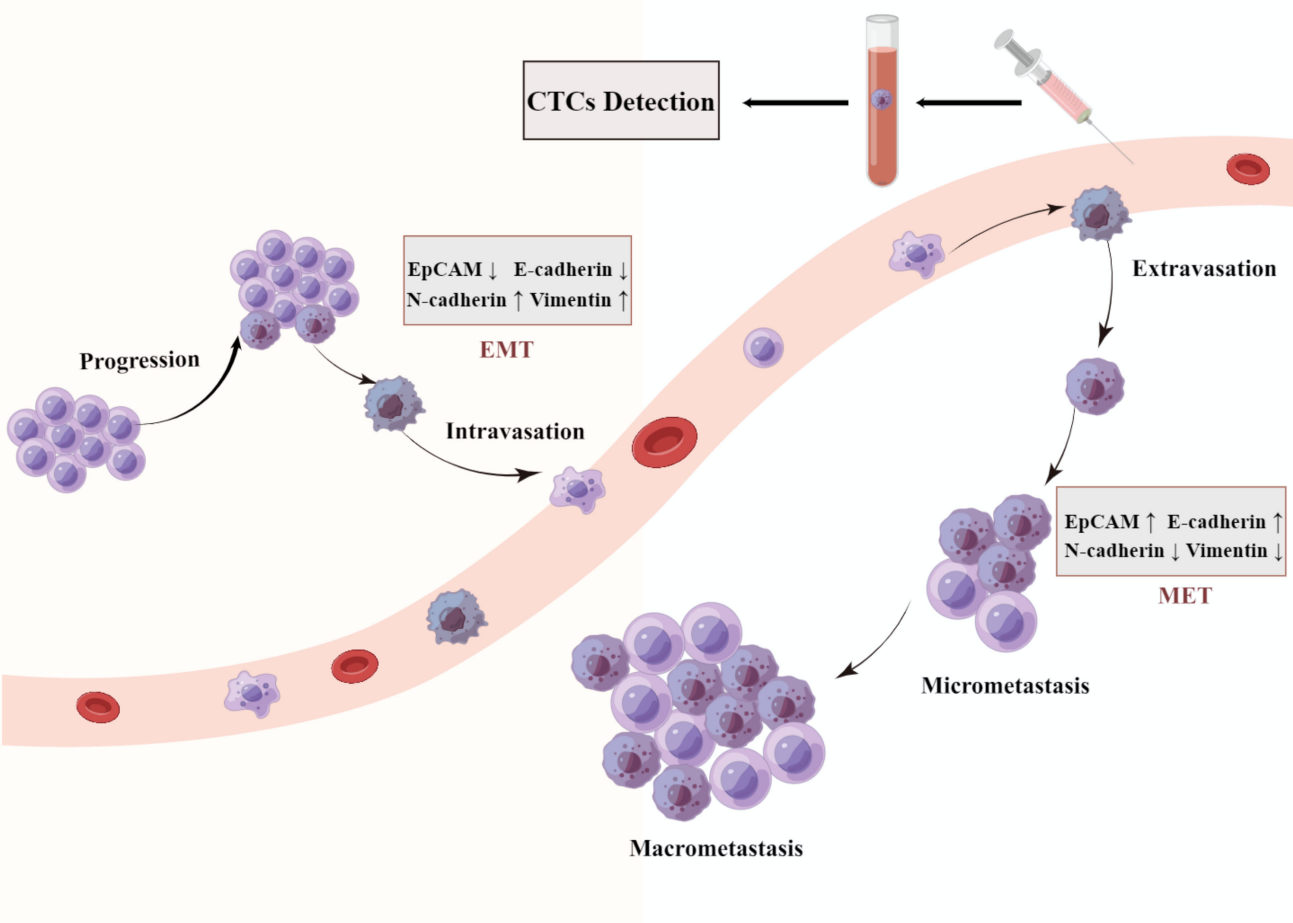
A schematic representation of epithelial mesenchymal transition (EMT). Cancer cells detach from the basement membrane and intravasate to the nearby blood vessels as CTC and travel through blood vessels to a secondary site in a process described as metastasis. They get lodged in different organs by a process termed as mesenchymal epithelial transition (MET).

Biological methods capture CTCs based on cellular immune characteristics that specific antibodies or ligands target to the antigens presented on the cell membrane of CTCs. The affinity ligands or antibodies are immobilized on microdevices or magnetic beads to bind with antigens on the cell membrane of CTCs, which achieve efficient and high-purity enrichment (36). Biological methods can be divided into two subcategories: positive techniques and negative techniques. Cellsearch system is the most representative of positive enrichment methods, which is only approved by the US Food and Drug Administration for the detection of CTCs (16). This system employs immunomagnetic beads coated with an antibody specifically against the epithelial Cell adhesion molecule (EpCAM) antigens on CTCs cell surface, and has already been applied in HCC (37–44). However, several types of tumor cells express low level of EpCAM, and some CTCs may lose the expression of EpCAM during the process of EMT (**Figure 1**). This largely limits the sensitivity of Cellsearch system for EpCAM negative tumor cells (45, 46). The negative enrichment method employs immunomagnetic beads coated with CD45 antibodies to bind the antigens on the surface of leukocytes, and then the binding leukocytes are removed with the beads (47, 48). This indirectly enriching CTCs method makes up for the limitation that the Cellsearch system is highly dependent on EpCAM. Additionally, many research studies show that CTCs detection with a single marker is inefficient. The combination of multiple markers and a physical enrichment method could increase the sensitivity and specificity of CTCs detection.

The most common method to identify CTCs are through immunofluorescence (IF), which provides the size and morphology of CTCs (49). Other identification methods includes flow cytometry (50), quantitative real-time PCR (qRT-PCR) (51) and immunofluorescence *in situ* hybridization (iFISH) (52). Now multiple detection methods have been used together to improve enrichment efficiency. Microfluidic chips achieve a high sensitivity and specificity in the separation of CTCs by the combination of cell size and immunoaffinity (53, 54). The CanPatrol system combines microfiltration and RNA *in situ* hybridization, which can simultaneously identify CTCs with EMT phenotype and has been widely used in various tumors including HCC (13, 22, 52, 55–61). A novel integrated strategy, subtraction enrichment (SE)-iFISH, improved the detection of HCC CTCs (30). EpCAM^+^ CTCs and EpCAM^−^ CTCs, each with different ploidy of chromosome 8, were effectively detected in patients with hepatobiliary malignances.

## EMT and CTCs

Many evidence shows a strong relationship between EMT and CTC. EMT is a dynamic process in which the epithelial phenotype cells transition into mesenchymal phenotype cells with the downregulation of epithelial markers (E-cadherin, EpCAM) and upregulation of mesenchymal markers (N-cadherin, vimentin) (62, 63). EMT is critical for embryonic development and tissue repair under physiological conditions, however, it is also thought to play a crucial role in tumor progression by promoting tissue infiltration and metastases (64, 65). EMT is a complex process involving multiple signaling pathways and transcription factor regulation, the up-regulation of mesenchymal genes and suppression of epithelial genes lead to the transformation of cell morphology and acquisition of stronger invasive migration capability (66). Many changes in cellular shape and vitality, such as loss of cellular polarity, the disappearance of intercellular adhesion, and the acquisition of enhanced motility, which promote cells to fall off from tumor tissues and invade into blood vessels (64, 67, 68). EMT process facilitates the secretion of matrix metalloproteinase (MMP), which degrades the extracellular matrix to cause cell migration, tissue invasion and blood vessel infiltration (69). EMT is believed to play an important role in tumor occurrence and progression. The studies have demonstrated that the expression of EMT markers is associated with an aggressive malignant phenotype and with poor prognosis in cancer patients (65, 67, 70).

Recent studies found that tumor cells undergoing EMT are associated with the acquisition of stem cell characteristics (71), immunosuppression (72), resistance to radiotherapy (73) and chemotherapy (74, 75). A recent study in patients with HCC reported that the presence of mesenchymal phenotype CTCs was significantly correlated with high AFP levels, multiple tumors, advanced TNM stages, presence of embolus or micro-embolus, and earlier recurrence (57). Another study showed that mesenchymal phenotype CTCs were an independent risk factor for early recurrence, indicating a shorter postoperative disease-free survival in patients with HCC (60).

EMT promotes the spread of cancer cells from the primary tumor into the blood. Upon arrival at a suitable secondary location, CTCs undergo the MET transformation from the non-proliferative or low-proliferative migratory phenotype to a proliferative type, resulting in distant metastasis (64, 67, 76). To form micro-metastases or develop into metastatic cancers, CTCs are considered to have a corresponding colonization ability in the blood circulation (77, 78). The hypothesis proposes that there may be a reversible switch between epithelial-mesenchymal transition (EMT) and mesenchymal-epithelial transition (MET) (79, 80). Contrary to the physiological process of EMT, mesenchymal phenotype cells transform into epithelial phenotype cells by recovery of intercellular adhesion and reduced mobility during MET, providing the ability of distant colonization and establishment of metastases.

## Biomarkers of CTCs in HCC

The biomarkers only expressed on the surface of liver tumor cells and rarely or not expressed in blood cells, which are used for CTCs detection in HCC patients. The biomarkers include epithelial markers, EMT-related markers, hepatocytes and HCC specific markers, and cancer stem-cell markers, as shown in **Table 1**.

**Table 1:** Summary of clinical application of CTCs in HCC.

CTC Marker	Method	Specimen	Main finding
***Epithelial Marker***			
**EpCAM**	CellSearch system	59 HCC; 19 BLD	CTCs were detected in 18/59 HCC patients and 1/19 in control patients. Patients with the presence of CTCs had shorter OS. Schulze et al., 2013 (38)
**EpCAM**	Negative enrichment+ qRT-PCR	122 HCC; 120 BLD	Preoperative CTC levels showed prognostic significance in HCC patients with surgical treatment. Combined with the AFP level, the AUC was 0.857 with a sensitivity of 73.0% and specificity of 93.4%. Guo et al., 2014 (51)
**EpCAM**	Magnetic separation+ IF	42 HCC; 10 BLD; 10 HV	Shedding of tumor cells during TACE did not affect the time to progression of HCC patients. Fang et al., 2014 (81)
**EpCAM**	CellSearch system	20 HCC; 9 BLD	CTCs were detected in 7 of 20 HCC and 0 of 9 BLD. The presence of CTCs was associated with AFP levels and vascular invasion. Kelley et al., 2015 (39)
**EpCAM**	CellSearch system	57 HCC	HCC patients with CTCs before the operation had a higher risk of recurrence and shorter RFS. Felden et al., 2017 (40)
**EpCAM**	CellSearch system	139 HCC	The increased postoperative CTC counts were significantly associated with the macroscopic tumor thrombus status, shorter DFS and OS. Yu et al., 2018 (41)
**EpCAM**	CellSearch system	309 HCC	Preoperative CTC counts were correlated with microvascular invasion, and patients with positive CTC should have enough surgical margins to protect against early recurrence. Zhou et al., 2020 (42)
**EpCAM**	CellSearch system	344 HCC	CTC-positive patients treated with adjuvant TACE had lower early recurrence, longer OS and time to recurrence. Wang et al., 2020 (43)
**EpCAM**	CellSearch system	197 HCC	The postoperative CTC counts ≥3 were associated with postoperative extrahepatic metastases and shorter median overall survival. Sun et al., 2020 (44)
***EMT related Marker***			
**Twist, Vimentin, ZEB1, ZEB2, Snail, Slug, ASGPRs, E-cadherin**	Magnetic separation+ IF	60 HCC	CTCs were detected in 46/60 HCC patients. Co-expression of Twist and vimentin in CTCs was closely correlated with portal vein tumor thrombus, TNM classification and tumor size. Li et al., 2013 (82)
**EpCAM, Vimentin, CK8/18/19**	CanPatrol system	40 HCC; 124 other cancers; 27 HV	CTCs were detected in 107/164 different cancer patients. The presence of mesenchymal CTCs tended to occur in patients with metastatic stages in different types of cancers. Wu et al., 2015 (22)
**EpCAM, Vimentin, CK8/18/19, Twist**	CanPatrol system	33 HCC; 10 HV	Epithelial-mesenchymal-mixed CTCs play an important role in EMT transition in HCC, mixed CTCs might be a vital factor for intrahepatic metastasis, and mesenchymal CTCs had the potential to be a predictor of extrahepatic metastasis. Liu et al., 2016 (56)
**EpCAM, Vimentin, CK8/18/19, Twist, E-cadherin, AKT2, Snail,**	CanPatrol system	195 HCC	CTCs were present in 95% of HCC patients. Mesenchymal and hybrid CTCs were correlated with ages, BCLC stages, metastasis, AFP levels and recurrence. Chen et al., 2017 (13)
**EpCAM, Vimentin, CK8/18/19, Twist**	CanPatrol system	165 HCC	CTCs were present in 70.9% of HCC patients. The presence of mesenchymal CTCs was significantly correlated with high AFP levels, multiple tumors, advanced TNM and BCLC stage, presence of embolus or micro-embolus, and earlier recurrence. Ou et al., 2018 (57)
**EpCAM, CK8/18/19**	CanPatrol sytem	80 HCC; 10 HV	Twist+ CTCs were detected in 54/80 HCC patients. The ratios of Twist+ CTCs were correlated with advanced stage, rate of metastasis, recurrence and mortality. the prognostic evaluation of Twist+ CTCs was better CTCs alone. Yin et al., 2018 (58)
**EpCAM, Vimentin, E-cadherin, Twist, CK8/18/19, BCAT1**	CanPatrol system	112 HCC; 12 HBV; 20 HV	CTCs were present in 90.18% of HCC patients. Preoperative mesenchymal-CTC percentage ≥2% was closely correlated with early recurrence, lung metastasis and multi-intrahepatic recurrence. Qi et al., 2018 (59)
**EpCAM, Vimentin, E-cadherin, CK**	CellSearch system and qRT-PCR	73 HCC	CTCs and circulating tumor micro-emboli burden in hepatic veins and peripheral circulation predicted postoperative lung metastasis and intrahepatic recurrence, respectively. Sun et al., 2018 (83)
**EpCAM, Vimentin, CK8/18/19, Twist**	CanPatrol system	62 HCC	Mesenchymal CTCs and portal vein tumor thrombus were independent risk factors for early recurrence. Patients with positive mesenchymal CTCs had significantly shorter postoperative disease-free survival. Wang et al., 2018 (60)
**EpCAM, Vimentin, CK8/18/19, Twist**	CanPatrol system	113 HCC; 57 BLD	All types of CTCs in patients with HCC were significantly more numerous than in BLD group patients. The use of total CTCs was more effective than AFP for the diagnosis of HCC, the combination of total CTCs and AFP could promote diagnostic sensitivity. Cheng et al., 2019 (61)
**CK, CD45**	Tapered slit platform+ IF	105 HCC; 132 BLD	The changes in CTCs count before and after surgery was defined as ΔCTC, and the increased ΔCTC was significantly associated with recurrence. Ha et al., 2019 (84)
**Vimentin, Twist**	CanPatrol system	261 HCC	The combination of PA-TACE and hepatic resection showed improved RFS and OS than hepatic resection alone for mCTC-positive patients. Zhang et al., 2021 (52)
**CK, CD45**	MCA system + IF	31 HCC; 14 BLD; 7 HV	The ratio of positive CTCs in HCC was higher than that in BLD. The enumeration of CTCs was associated with tumor stage and the presence of CTCs (≥10) tended to significantly reduce the cumulative survival. Takahashi et al., 2021 (85)
**EpCAM, CK19, p-CK**	ChimeraX^®^-i120 platform	193 HCC	Postoperative CTC count ≥1 was correlated with tumor recurrence after LTx, and postoperative serial CTC detection could be applied in surveillance for recurrence. Wang et al., 2021 (49)
***Liver and HCC specific markers***			
**ASGPR, Hep Par 1**	Magnetic separation+ IF	85 HCC; 37 BLD; 20 HV; 14 other cancers	CTCs were present in 69/85 HCC patients, and no CTCs were detected in healthy, BLD or other cancer groups. The detection rate and enumeration of positive CTCs were significantly associated with tumor size, portal vein tumor thrombus and TNM stage. Xu et al., 2011 (86)
**ASGPR, CPS1, P-CK**	Magnetic separation+ IF	27 HCC	CTCs were identified in 89% of HCC patients by this method, and no CTCs were found in the other test subjects. Li et al., 2014 (87)
**ASGPR, CPS1**	negative enrichment+ IF	32 HCC; 40 BLD; 20 HV; 17 other cancers	CTCs with positive ASGPR and CPS1 were detected in 91% of HCC patients, and no CTCs were found in healthy volunteers, BLD group and other cancer patients. Liu et al., 2015 (47)
**CK, EpCAM, AFP, GPC3, DNA-PK**	imaging flow cytometry method	69 HCC; 31 controls	CTCs were detected in 45/69 HCC patients and 0/31 controls, the enumeration of positive CTCs was correlated with tumor size, portal vein thrombosis and shorter median survival. Ogle et al., 2016 (88)
**TP53**	CanPatrol system	42 HCC	The postoperative CTC counts (> 2) and changes in CTC counts between preoperation and postoperation could be independent prognostic indicators for PRS in patients with HCC. Ye et al., 2018 (89)
**ASGPR, GPC3**	magnetically assisted surface-enhanced Raman scattering	8 HCC; 5 HV; 5 other cancers	The platform with dual labeling of ASGPR and GPC3 had an effective ability in detecting HCC CTCs with a small number of peripheral blood samples in clinical diagnosis. Pang et al., 2018 (90)
**GPC3**	immunomagnetic positive enrichment +flow cytometry	85 HCC	The preoperative GPC3-positive CTCs were a risk factor for microscopic portal vein invasion and poor prognosis. Hamaoka et al., 2019 (50)
**EpCAM, ASGPR**	Microfluidic Synergetic-Chip	45 HCC	The platform with dual labeling of EpCAM and ASGPR had an effective ability in detecting HCC CTCs. CTCs were identified in 100% of HCC patients. Total CTCs and non-epithelial CTCs were associated with advanced stage and malignant progression. Zhu et al., 2020 (53)
***Stem-cell markers***			
**CD90(+), CD44(+)**	Multicolor flow cytometry	82 HCC	Circulating CSCs > 0.01% was correlated with intrahepatic recurrence and extrahepatic recurrence, also associated with lower RFS and OS. Fan et al., 2011 (91)
**EpCAM, CD133, ABCG2**	CellSearch system	123 HCC	CSC biomarkers CD133 and ABCG2 were displayed in EpCAM positive CTCs. Sun et al., 2013 (92)
**GPC3, CS, CD44, Hep Par-1**	The Labyrinth Chip+IF	42 HCC	CTCs were detected in 88.1% of HCC patients and CTCs with the expression of CD44 were observed in 71.4% of HCC patients. CTCs with GPC3, CS and HepPar-1 markers had a cancer stemness phenotype. Wan et al., 2019 (93)
***Drug therapy monitoring***			
**pERK, pAkt**	negative enrichment+ IF	109 HCC	HCC patients with pERK^+^/pAkt^−^ CTCs were most sensitive to sorafenib. The proportion of pERK^+^/pAkt^−^ CTCs was significantly correlated with shorter PFS, and could be an independent predictive factor in HCC patients treated with sorafenib. Li et al., 2016 (48)
**CK, PD-L1**	NanoVelcro Chip	87 HCC; 7 BLD; 8 HV	PD-L1+ CTCs were identified in 8.2% of early-stage patients, 54.5% of locally advanced and 93.8% of metastatic patients. HCC patients with PD-L1+ CTCs had favorable treatment responses when receiving anti-PD-1 therapy. Winograd et al., 2020 (94)

### Epithelial markers

EpCAM is one of the most commonly used epithelial markers on the cell membrane, and is widely used for isolating CTCs from peripheral blood. EpCAM^+^ CTCs enriched by CellSearch system were detected in a cohort of 29 patients, including 20 in the HCC group and 9 in the control group (39). A total of 7/20 (35%) HCC patients had more than two CTCs per 7.5 mL in peripheral blood. Although no patients with non-malignant liver diseases were detected with CTCs, the presence of CTCs was associated with AFP level and vascular invasion. Sequence analysis of CTCs DNA showed that low frequency variants exist in EpCAM^+^ CTCs. Another study consists of 197 HCC patients who underwent curative surgical resection and show that EpCAM^+^ CTCs counts (≥3) were associated with postoperative extrahepatic metastases and shorter median overall survival. The HCC patients with high number of CTCs need more careful surveillance in early interventions (44). EpCAM-based enrichment methods are not able to be used in the enrichment of CTCs which express low level of EpCAM (95) or lose expression of EpCAM during the process of EMT (96, 97).

### EMT-related markers

According to the expression patterns during EMT process, biomarkers are largely categorized into epithelial markers and mesenchymal markers. Epithelial markers contain E-cadherin, EpCAM and CK8/18/19. Mesenchymal markers include N-cadherin, vimentin and some transcription factors such as ZEB1, ZEB2, twist, slug and Snail1 (98). Based on microfiltration enrichment and RNA *in situ* hybridization, the CanPatrol system captures CTCs and classifies them into epithelial phenotype, mesenchymal phenotype and mixed phenotype (52). A retrospective cohort study consists of 165 HCC patients who underwent curative surgical resection, and show that more than two preoperative CTCs were present in 117/165 (70.9%) of HCC patients and the elevated CTC counts were correlated with poor clinicopathological features (57). The presence of preoperative mesenchymal CTCs was significantly correlated with multiple tumors, high AFP levels, presence of embolus or micro-embolus, advanced TNM and BCLC stage, and earlier recurrence. Another recent study consists of 112 HCC patients who underwent surgical resection, 12 HBV patients and 20 healthy volunteers (59). A total of 101/112 (90.18%) HCC patients and 2/12 (16.67%) HBV patients were detected with CTCs. Two of HBV patients who were isolated CTCs and then were detected with small HCC within five months, nevertheless, none of the healthy volunteers were isolated CTCs. Preoperatively total CTCs or mesenchymal CTCs were closely correlated with early recurrence, lung metastasis and multi-intrahepatic recurrence. Mesenchymal positive CTCs are a high risk of early recurrence. The combination of epithelial and mesenchymal markers increases the sensitivity and specificity of CTCs detection. CTCs expressing EMT-related markers are significantly correlated with clinicopathological features, which allows adequate stratification for HCC patients’ management.

### Hepatocytes and HCC specific markers

Serum AFP is a predictor of prognostic evaluation because the high level of AFP is closely associated with HCC recurrence and metastasis (2, 99). AFP is common biomarker for HCC screening, however, in early HCC the detection rate of AFP is only 25-65% (100). There is an urgent need of HCC specific biomarkers in early detection. A number of hepatocytes and HCC specific markers, including sialoglycoprotein receptor (ASGPR), hepatocyte paraffin 1 (Hep Par 1), and glypican-3 (GPC3), show a diagnostic and prognostic value (47, 50, 53, 86–88, 90).

A study consist of 85 HCC patients, 37 patients with benign liver diseases, 14 patients with other cancers, and 20 healthy volunteers (86). The immunomagnetic enrichment of ASGPR^+^ CTCs and subsequent identification by immunofluorescence staining with Hep Par 1 antibody were illustrated to be an effective strategy to identify CTCs from peripheral blood of HCC patients. ASGPR is a protein expressed in the outer membrane of hepatocytes and HCC cells. Hep Par 1 is a protein expressed on the mitochondrial membrane of hepatocytes. By this method, CTCs were present in 69/85 (81%) HCC patients, and no CTCs were detected in benign liver diseases, healthy volunteers, or other cancer groups. The counts of CTCs were significantly associated with tumor size, portal vein tumor thrombus and TNM stage. Another study consists of 32 HCC patients, 40 patients with benign liver diseases, 20 healthy volunteers and 20 healthy volunteers (47). The negative enrichment method with CD45 antibodies to remove leukocytes and subsequent identification using a combination of ASGPR and CPS1 antibodies were conducted to identify CTCs from peripheral blood of HCC patients. By this strategy, CTCs with positive ASGPR and CPS1 were detected in 29/32 (91%) of HCC patients, nevertheless, no CTCs were found in healthy volunteers, benign liver diseases and other cancer groups. Recently, Zhu and his colleagues designed and developed a microfluidic Synergetic-Chip, which combined physical size separation and immunological recognition (53). EpCAM and ASGPR antibodies were respectively coated in two parallel channels to capture CTCs, subsequently, enriched CTCs were identified by immunofluorescence staining. By this method, EpCAM or ASGPR positive CTCs were identified in 45/45 (100%) of HCC patients, and the sensitivity and specificity achieve 97.8% and 100%. The specific markers combined with EpCAM were also used and proved to be efficient in CTCs detection (53, 101).

### Cancer stem-cell markers

Cancer stem cells (CSCs) are a specific sub-population of cancer cells in tumor tissue, which play an important role in the occurrence and metastasis of carcinoma. The origin of cancer stem cells remains controversial. One of the possible origins is the transformation through mutation and de-differentiation (102). These phenotypic changes are primarily the result of fundamental changes in gene expression concomitant with a diminished transcription of the relevant liver-specific genes, and can be interpreted as a ‘de-differentiation’ of hepatocytes. During the de-differentiation process, the overexpression of oncogenes and inactivation of tumor suppressor genes lead to uncontrolled excessive proliferation of cells and expression of stem cell markers (103). Currently, it is difficult to distinguish CTCs and CSCs due to the lack of a suitable markers. HCC cells and liver cancer stem cells are also derived from the de-differentiation of mature hepatocytes (103–105). Liver cancer stem cells are a unique subset of hepatocellular carcinoma cells with stem cell features and contribute to the disease recurrence, drug resistance and death (98).

Circulating CSCs are a few subpopulations of CTCs, which exhibit unlimited self-renewal and proliferation potential. They are associate with increased aggressiveness and poor prognosis (106). Some CTCs of peripheral blood of HCC patients expressed stem cell markers such as CD44, CD90, and CD133 (91–93). CD133^+^ CD44^+^ CSCs had been proved to be associated with elevated serum AFP, serum transaminases and poorer prognosis in HCC patients (107). Based on the detection of CD90^+^ CD44^+^ CSCs by multicolor flow cytometry, the number of preoperative CSCs in patients with recurrence were higher compared with the patients without recurrence (91). Patients with CD90^+^ CD44^+^ CSCs had lower RFS and OS. Thus, preoperative CSCs can be used to predict post-hepatectomy recurrence of HCC. EpCAM1^+^ CTCs of peripheral blood of HCC patients showed high tumorigenic capacity and low apoptotic tendency and expressed CSCs markers CD133^+^ and ABCG2^+^ (92). Through physical enrichment by Labyrinth chip and immunofluorescence staining using GPC3, CD44, and HepPar-1 antibodies, CTCs were detected in 37/42 (88.1%) of HCC patients, and CD44^+^ CTCs were identified in 30/42 (71.4%) of HCC patients (93). The detection rate of CD44^+^ CTCs was significantly higher in patients with advanced stages than those with early stages.

## Clinical application of CTCs in HCC

**Figure 2:**
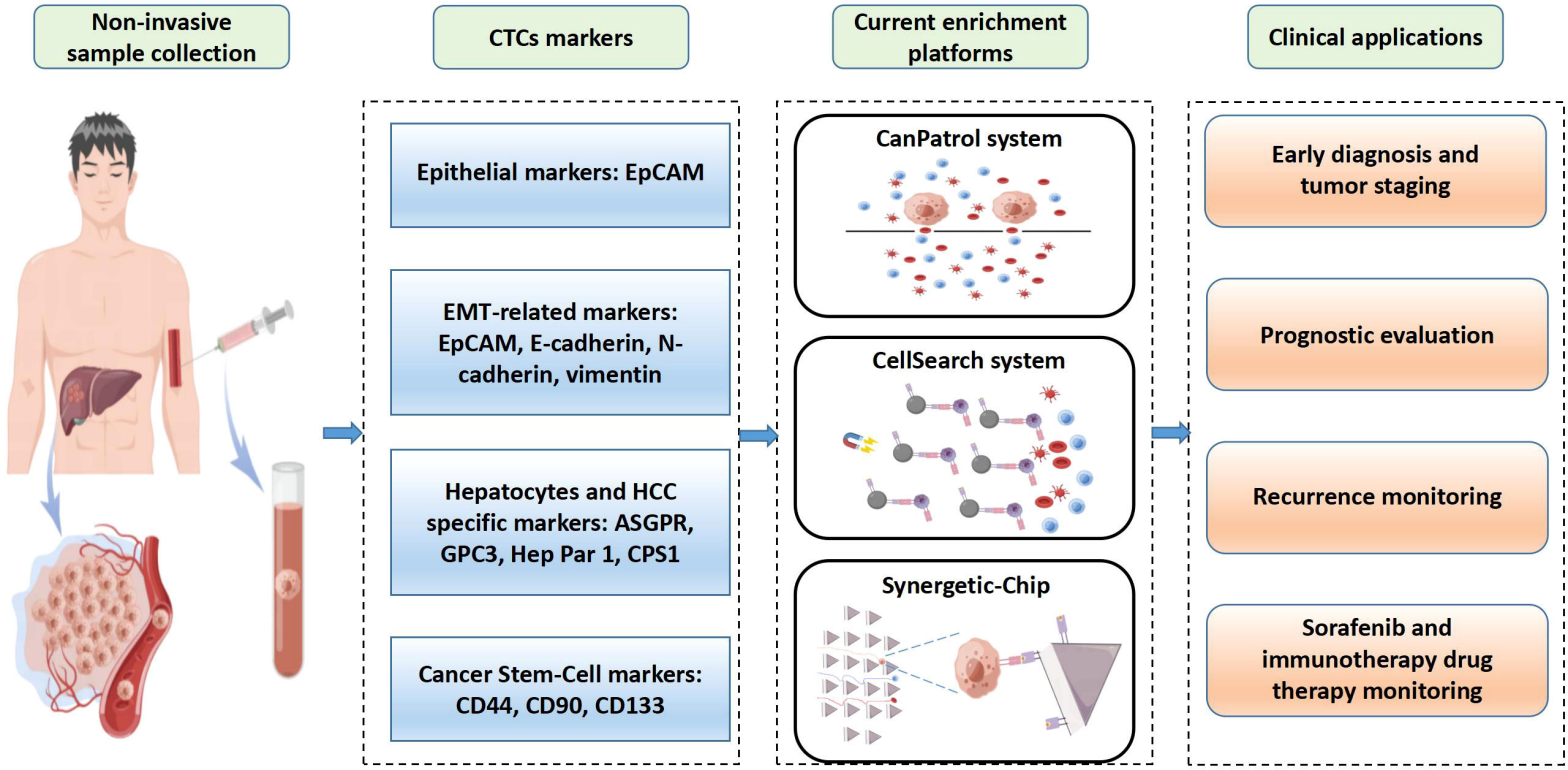
Overview of clinical applications of circulating tumor cells (CTCs) in hepatocellular carcinoma (HCC). CTCs are obtained from patients’ blood samples in a non-invasive way. HCC CTCs are primarily isolated based on their unique biological markers. CellSearch is the only FDA-approved system for CTCs detection used clinically. CanPatol and CTC-chip are other CTCs detection systems. CTCs represent an independent factor for early diagnosis and tumor staging, prognostic evaluation, recurrence monitoring, and drug therapy monitoring.

CTCs may transfer through hematogenous metastasis in the early stage, so some HCC patients have distant metastasis before receiving treatment, which leads to a great challenge for HCC management. Therefore, early diagnosis of HCC is the key to receiving the effective treatment. Accumulated evidence indicates that CTCs detection is of great significance for early diagnosis HCC. Related detection markers, methods and clinical application of CTCs are shown in **Figure 2**.

### Diagnosis and tumor staging

Many evidence indicates that CTCs detection with EMT-related markers are useful for early diagnosis and staging. EpCAM^+^ CTCs were detected in 18/59 (30.5%) HCC patients and 1/19 (5.3%) individuals in the control group. The CTCs detection rates of patients with different BCLC stages were significant differences: stages A 1/9 (11.1%), B 6/31 (19.3%) and C 11/19 (57.9%) (38). CTCs were detected in 101/112 (90.12%) of HCC patients, even at an early stage (59). Patients with late BCLC stage had a higher mesenchymal phenotype CTCs counts than those in early stage. Mesenchymal phenotype CTCs counts (≥1) could be used to distinguish BCLC stage in HCC patients (61). The combination of total CTCs and AFP improve the sensitivity and specificity of 61.95% and 89.47%, respectively. Twist^+^ vimentin^+^ CTCs were detected in 33/46 (69.6%) of HCC patients. The CTCs detection rates were closely associated with tumor size, portal vein tumor thrombus, and TNM classification (82). CTCs were detected in 185/195 (95%) of HCC patients. The counts, mesenchymal and mixed phenotype of CTCs were correlated with the clinicopathologic features, such as ages, BCLC stages and AFP levels (13, 22, 52, 57, 58, 61).

HCC specific markers are efficient to detect CTCs clinically. CTCs were identified in 24/27 (89%) HCC patients by specific markers ASGPR, CPS1 and P-CK, and no CTCs were detected in other test subjects (87). ASGPR^+^CPS1^+^ CTCs were detected in 29/32 (91%) of HCC patients, and no CTCs were found in healthy volunteers, benign liver diseases and other cancers groups (47). ASGPR^+^ EpCAM^+^ CTCs were detected in 45/45 (100%) of HCC patients (53). Some study showed that CTCs counts through HCC specific markers or combination with other markers were correlated with tumor size, differentiation status, portal vein tumor thrombus and TNM stage (50, 53, 86, 88).

### Prognostic evaluation and recurrence monitoring

The prognosis of HCC is closely related to the clinical stage. The common clinical prognostic models of HCC include the BCLC staging system and TNM staging system. BCLC staging system is based on tumor burden, liver function and physical status. The parameters of tumor burden are composed of the number and diameter of tumor, portal vein invasion and extrahepatic spread (108). TNM staging is evaluated by the number and diameter of tumors, vascular invasion, adjacent organs invasion, regional lymph nodes metastases and distant metastases (109, 110). Studies have shown that the counts of CTCs are associated with the parameters of BCLC staging and TNM staging in HCC patients (13, 52, 53, 59). Analysis of the CTCs by RNA *in situ* hybridization (RNA-ISH) revealed that positive rate of CTCs was 83.6% (46/55) and 96.5% (55/57) in patients with BCLC stage 0–A and stage B–C tumors (59). A meta-analysis study of twenty-three published studies showed that CTC positivity were also significantly associated with TNM Stage (RR 1.30, 95% CI: [1.02-1.65]; p=0.03), tumor size (RR 1.36, 95% CI: [1.09-1.69]; p=0.006), vascular invasion (RR 1.99, 95% CI: [1.43-2.77]; p<0.0001), portal vein tumor thrombus (RR 1.73, 95% CI: [1.42-2.11]; p=0.0001), serum AFP level (RR 2.05, 95% CI: [1.18-3.54]; p=0.01), suggesting a strong prognostic value of CTC in HCC (111).

The counts of CTCs make a difference in peripheral blood of HCC patients before and after treatment. Thus, CTCs detection is used to monitor the response of therapy and help clinicians to adjust their treatment plans. Recent study reported the relationship between ΔCTC and recurrence of HCC after hepatectomy. ΔCTC is defined as the changes of CTCs counts after surgery. The positive ΔCTC was correlated with higher recurrence and lower survival in HCC patients after surgical liver resection (84). HCC patients with postoperative EpCAM^+^ CTCs counts < 2 had significantly longer disease-free survival and overall survival than the patients with CTCs counts ≥2, suggesting increased postoperative EpCAM^+^ CTCs counts were related to a poor clinical outcome after surgical liver resection (41). Some studies provided evidence that mesenchymal phenotype CTCs could predict early recurrence, metastasis, shorter disease-free survival and overall survival (13, 52, 56, 57, 59, 82, 83). EMT-related phenotype CTCs play an important role in predicting metastasis and recurrence. A study reported that Twist^+^ CTCs were associated with higher metastasis, recurrence and mortality rate (58). Another study found that mesenchymal phenotype CTCs were independent risk factors for early recurrence. HCC patients with postoperative mesenchymal phenotype CTCs counts ≥1 had a shorter disease-free survival after surgery (60). The preoperative GPC3^+^CTCs were significantly correlated with the clinical outcome of HCC patients. GPC3^+^ CTCs counts >5 were an independent risk factor for microscopic portal vein invasion. HCC patients with GPC3^+^ CTCs counts >5 had lower disease-free survival and overall survival after hepatectomy (50). The preoperative CD90^+^ CD44^+^ CSCs were proved to be associated with intrahepatic and extrahepatic recurrence of patients after surgical resection. HCC patients with CSCs > 0.01% (the optimal cut-off point for gating percentages of circulating CSCs by calculating the AUROC) had lower disease-free survival and overall survival (91). HCC patients with PD-L1^+^ CTCs had shorter overall survival than those patients without PD-L1^+^ CTCs (94).

### Drug therapy monitoring

Drug resistance is also an important factor affecting clinical prognosis. The acquired drug resistance of HCC cells leads to poor effects of systemic therapy (112). Sorafenib is a small molecule multi-kinase inhibitor with multiple antitumor effects and is the first-line targeted drug for the treatment of HCC in the advanced stage (113). Numerous studies have shown that the inactivation of Ras/Raf/ERK pathway and the activation of PI3K/Akt/mTOR pathway plays a crucial role in sorafenib resistance (114–117). CTCs are released from tumor tissue to the bloodstream and carry drug resistance information of the primary tumor or metastases, therefore pERK/pAkt phenotype of CTCs can be used to monitor the therapeutic effect and drug resistance of sorafenib (48). A study reported the association between pERK^+^ pAkt^−^ CTCs and the sensibility of sorafenib in HCC patients (48). During sorafenib treatment, the CTC counts showed a marked decrease in patients with pERK^+^ pAkt^−^ CTCs. HCC patients with pERK^+^ pAkt^−^ CTCs were sensitive to sorafenib-targeted therapy, whereas patients with other phenotype CTCs showed resistance to sorafenib therapy. So, they suggested the percentage of pERK^+^ pAkt^−^ CTCs could serve as an independent predictive factor for HCC patients treated with sorafenib.

The programmed cell death protein 1 (PD-1) and its ligand (PD-L1) axes is an inhibitory immune checkpoint that is involved in suppressing antitumor immunity. The overexpression of PD-L1 of tumor cells binding to PD-1 on the surface of T cells can induce T-cell anergy or apoptosis, which enables evasion of immune-mediated tumor surveillance (112). Inhibitors targeting PD-1 or PD-L1 can restrain this pathway and restore antitumor immunity (118). The advanced melanoma patients with PD-L1^+^ CTCs showed a good response to PD-1 inhibitor immunotherapy, whereas those patients with the PD-L1^−^ CTCs exhibited drug resistance (119). PD-L1^+^ CTCs which were predominantly found in advanced-stage patients could accurately discriminate early-stage from advanced-stage HCC patients (94). Subsequently, they followed up 10 HCC patients who received immunotherapy (PD-1 blockade), and found that 5 patients with PD-L1^+^ CTCs had a good response to immunotherapy, and only 1 of 5 non-responders had PD-L1^+^ CTCs. CTCs with specific phenotypes have the potentials to predict drug resistance to targeted therapy and immunotherapy, which provide clinical application in guiding individualized medicine.

## Conclusions

The detection of CTCs is of great value in the early diagnosis and tumor staging, evaluation of recurrence and prognosis, and even prediction of drug resistance of targeted therapy and immunotherapy in patients with HCC. However, CTCs detection is not widely used in the clinical application of HCC due to several limitations, such as lack of standardized technical procedures, limitations of detection efficiency, extremely rare of CTCs in blood, and absence of external validation in many clinical studies. If technological advances could overcome the shortcomings of CTCs detection in the future, CTCs detection will be a breakthrough in HCC clinical management, especially in early diagnosis offering an opportunity for curative surgery before metastasis. EMT plays an important role in various pathophysiological processes of CTCs, such as tumor cells shedding and migrating, vascular invasion and distant implantation. More mechanisms of EMT in CTCs should be investigated, which is expected to provide new insight into HCC clinical management.

## Author contributions

YY, JD, JH, and BW reviewed the literature. YH and YY drafted the manuscript and designed the figures. YY and ZL revised the manuscript. All authors read and approved the manuscript.

## Funding

This work was supported by Guangdong Basic and Applied Basic Research Foundation (2021A1515011261).

## Conflict of interest

The authors declare that the research was conducted in the absence of any commercial or financial relationships that could be construed as a potential conflict of interest.

## Publisher’s note

All claims expressed in this article are solely those of the authors and do not necessarily represent those of their affiliated organizations, or those of the publisher, the editors and the reviewers. Any product that may be evaluated in this article, or claim that may be made by its manufacturer, is not guaranteed or endorsed by the publisher.

## Abbreviations

AFP, alpha-fetoprotein; ASGPR, sialoglycoprotein receptor; BCLC, Barcelona clinic liver cancer; CD44, cluster of differentiation 44; CD45, cluster of differentiation 45; CD90, cluster of differentiation 90; CD133, cluster of differentiation 133; CPS1, carbamoyl phosphate synthetase 1; CSCs, circulating cancer stem cells; CTCs, Circulating tumor cells; EMT, Epithelial-mesenchymal transformation; EpCAM, epithelial Cell adhesion molecule; GPC3, glypican-3; HCC, Hepatocellular carcinoma; Hep Par 1, hepatocyte paraffin 1; IF, immunofluorescence; iFISH, immunofluorescence *in situ* hybridization; MET, mesenchymal-epithelial transition; MMP, matrix metalloproteinase; pAkt, phosphorylated protein kinase B; PD-L1, programmed cell death ligand 1; pERK, phosphorylated extracellular signal-regulated kinase; qRT-PCR, quantitative real-time PCR; TNM, tumor-node-metastasis; AUC, area under the curve; BLD, benign liver disease; CTCs, circulating tumor cells; DFS, disease-free survival; EMT, epithelial to mesenchymal transition; HCC, hepatocellular carcinoma; HV, healthy volunteers; IF, immunofluorescence; LTx, liver transplantation; OS, overall survival; PFS, progression-free survival; RFS, recurrence-free survival; TACE, transcatheter arterial chemoembolization; TNM, tumor-node-metastasis
